# Sex differences in brainstem structure volumes in patients with schizophrenia

**DOI:** 10.1038/s41537-023-00345-0

**Published:** 2023-03-18

**Authors:** Shintaro Aoyama, Hiroto Okuda, Natsu Furuzawa, Hirokage Yoneda, Daisuke Fujikane, Kentaro Takai, Ayumi Kuramitsu, Yukimasa Muto, Shunsuke Sugiyama, Toshiki Shioiri, Kazutaka Ohi

**Affiliations:** 1 School of Medicine, Gifu University, Gifu, Japan; 2 Department of Psychiatry, Gifu University Graduate School of Medicine, Gifu, Japan; 3 Department of General Internal Medicine, Kanazawa Medical University, Ishikawa, Japan

**Keywords:** Schizophrenia, Biomarkers

## Abstract

Patients with schizophrenia (SZ) display moderate reductions in brainstem volumes, including the midbrain, pons, superior cerebellar peduncle, and medulla oblongata. Here, we investigated alterations in brainstem volumes between SZ patients and healthy controls (HCs) stratified by sex. T1-weighted MRI brain scans were processed with FreeSurfer v6.0 in 156 SZ patients (61 males/95 females) and 205 HCs (133/72). Of the brainstem structures, pons volumes were significantly reduced, particularly in male SZ patients. The decreased pons volumes were correlated with lower levels of education but not duration of illness in male patients. These findings suggest that the reduction in pons volume in male patients might be occurred before or around the onset of the disorder.

## Introduction

The brainstem, which is located at the base of the brain, controls vital functions, such as the regulation of sleep/arousal, breathing, heart rate, and blood pressure, and subserves emotions and behavior^1,2^. The brainstem includes the midbrain, pons, superior cerebellar peduncle (SCP, which interconnects the pons and cerebellum), and medulla oblongata. A study found moderate reductions in these brainstem volumes in 1,044 patients with schizophrenia (SZ) compared to 2,079 healthy controls (HCs) (Cohen’s *d* = −0.10 for midbrain to −0.17 for pons)^3^. These decreased brainstem volumes may contribute to symptoms of SZ, such as impairments in sleep, autonomic functions and reflexes, although there were no significant associations between clinical symptoms and brainstem volumes in SZ patients^3^. Recent genome-wide association studies (GWASs) of midbrain, pons, SCP, and medulla oblongata volumes have indicated substantial heritability (*h*^*2*^ = 0.27 for SCP to 0.47 for midbrain and pons) and identified several genetic loci linked to brainstem volumes^3^. Moreover, a study found shared genetic loci between brainstem volumes and SZ^3^.

In addition to genetic factors and aging, there are sex differences in brainstem volumes, although these findings are not consistent across studies. Some studies have reported that males have larger brainstem volumes than females^4^, while others have reported no significant differences between the sexes or sex differences in the opposite direction^5,6^. Although the potential mechanisms underlying the sex differences are not yet fully understood, sex differences in brainstem volumes may cause sex differences in physiological function, behavior, and cognition. There is evidence for differences between males and females regarding the course and symptoms of SZ^7^. Males tend to develop SZ at an earlier age than females. Additionally, males may be more likely to experience a sudden onset of symptoms and negative symptoms. Considering the sex differences in the course and symptoms of SZ^7^, further research is needed to fully understand the relationship between brainstem volumes and SZ stratified by sex.

We hypothesized that alterations in brainstem volumes between SZ patients and HCs would be differed by sex, and the alterations in brainstem volumes would be related to clinical variables in SZ patients. In this study, we investigated alterations in four brainstem volumes between SZ patients and HCs stratified by sex.

## Methods

T1-weighted MRI brain scans using a Siemens 3 T Magnetom Trio, a Tim System (Siemens, Erlangen, Germany), were collected from 156 SZ patients (61 males/95 females; mean age±SD: 42.3 ± 13.3 years) and 205 HCs (133 males/72 females; 35.7 ± 13.6 years) (Supplementary Table 1). The sample information and details regarding sample collection, inclusion and exclusion criteria, diagnostic procedures, MRI sequences, and quality controls for imaging and segmentation have been described previously^8,9^. Briefly, participants were recruited from the Schizophrenia Non-Affected Relative Research Project (SNARP)^10^. The in/outpatients were diagnosed using the DSM-5. HCs without current or past contact with psychiatric services, psychiatric medication, or family history of neuropsychiatric disease within second-degree relatives were recruited. Subjects were excluded from the analysis if they had neurological or medical conditions that could affect the central nervous system. Written informed consent was obtained from all participants. This study was approved by the Research Ethical Committees of Gifu University and Kanazawa Medical University.

**Fig. 1:**
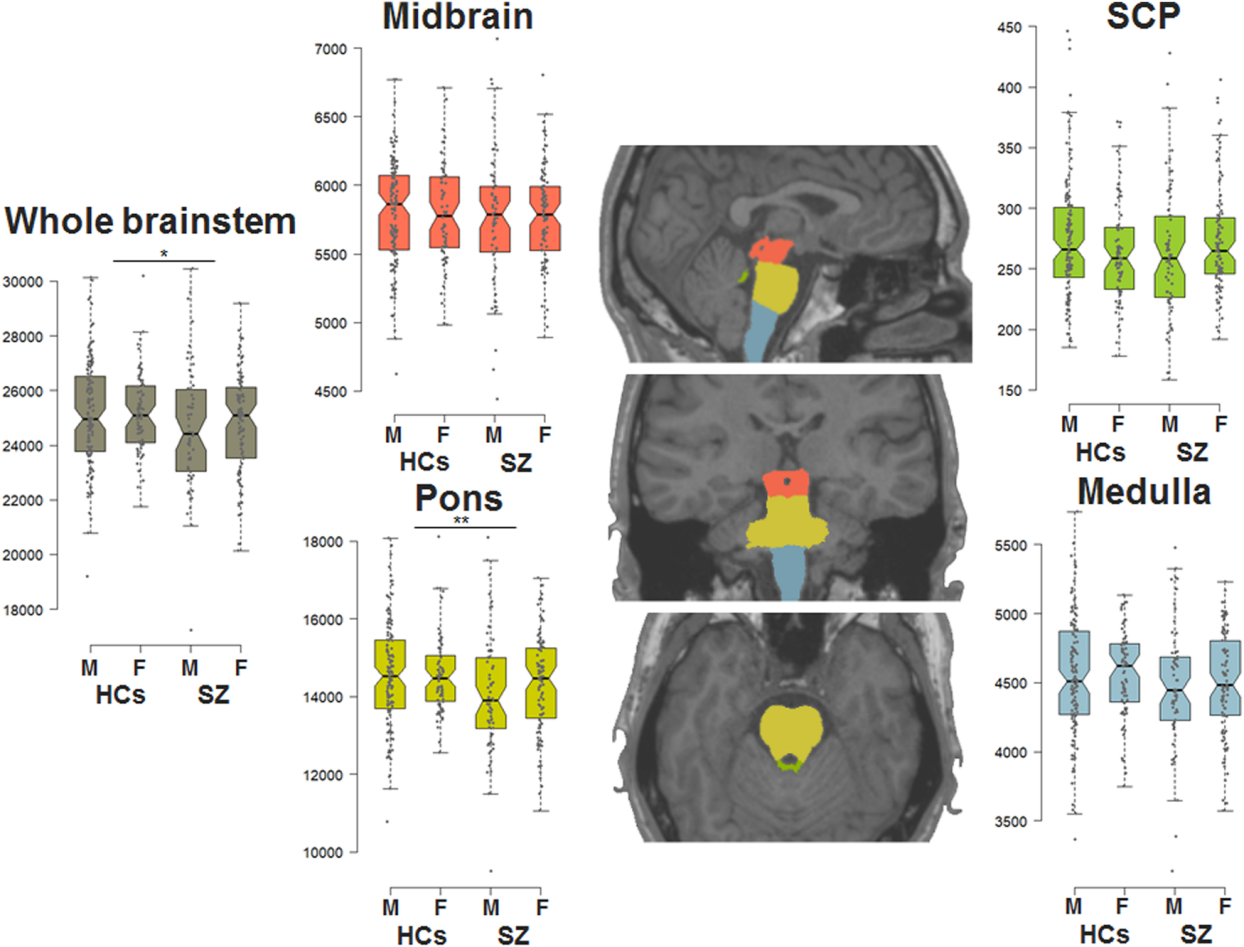
Alterations in four brainstem volumes (midbrain, pons, SCP, and medulla) between patients with schizophrenia and healthy controls. ^*^p < 0.05, ^**^p < 0.0125 (compared with HCs). SCP superior cerebellar peduncle, M male, F female, SZ schizophrenia, HCs healthy controls.

Because subjects with MRI abnormalities and images with motion or metal artifacts were screened out prior to inclusion in this study, there were no gross abnormalities in any of the subjects. Next, the T1-weighted images were fully automatically processed with FreeSurfer v6.0 with the default reconstruction procedure. Using the procedure, four brainstem structure volumes, the midbrain, pons, SCP, and medulla oblongata, were obtained (Fig. 1). Segmented brainstem structure was visually inspected, and there were no subjects with obviously poor segmentation. Differences in the brainstem volumes between case and control groups were analyzed using analysis of covariance (ANCOVA) with brainstem volumes as the dependent variables; case‒control status as an independent variable; and age, age-squared, (sex), and intracranial volume (ICV) as covariates. Sex was included as a covariate in the analysis for whole participants, but sex was not included as a covariate in the analysis stratifying sex. Additionally, partial correlations between brainstem volumes and clinical variables were investigated with age, age-squared, (sex), and ICV as covariates in SZ patients. The significance level was set at a two-tailed *p* < 0.0125 (*α* = 0.05/4 brainstem structures).

## Results

Male HCs had decreased brainstem volumes, except for medulla oblongata, compared with female HCs (*F*_*1,200*_ > 17.59, *p* < 0.05). Furthermore, male SZ patients had decreased midbrain (*F*_*1,151*_ = 15.77, *p* = 1.11 × 10^−4^), pons (*F*_*1,151*_ = 5.81, *p* = 0.017), and whole brainstem volumes (*F*_*1,151*_ = 7.25, *p* = 7.89 × 10^−3^) compared with female SZ patients. Of the four brainstem structures, pontine volumes in SZ patients were significantly smaller than those in HCs (Fig. 1, *F*_*1,355*_ = 6.62, *p* = 0.010). In contrast, although there were no significant differences in the other three brainstem structures between diagnostic groups (Fig. 1, p > 0.05), whole brainstem volumes consisting of all four structures were nominally decreased in SZ patients compared to HCs (*F*_*1,355*_ = 5.08, *p* = 0.025). Stratifying by sex, the differences in pons and whole brainstem volumes between diagnostic groups were prominent in male individuals (Fig. 1). Male SZ patients had decreased pons (*F*_*1,189*_ = 6.70, *p* = 0.010) and whole brainstem volumes (*F*_*1,189*_ = 5.50, *p* = 0.020) compared with male HCs, while there were no significant differences in brainstem volumes between female cases and control subjects (*p* > 0.05). Even after adjusting for handedness as covariates, these findings were not changed.

**Fig. 2:**
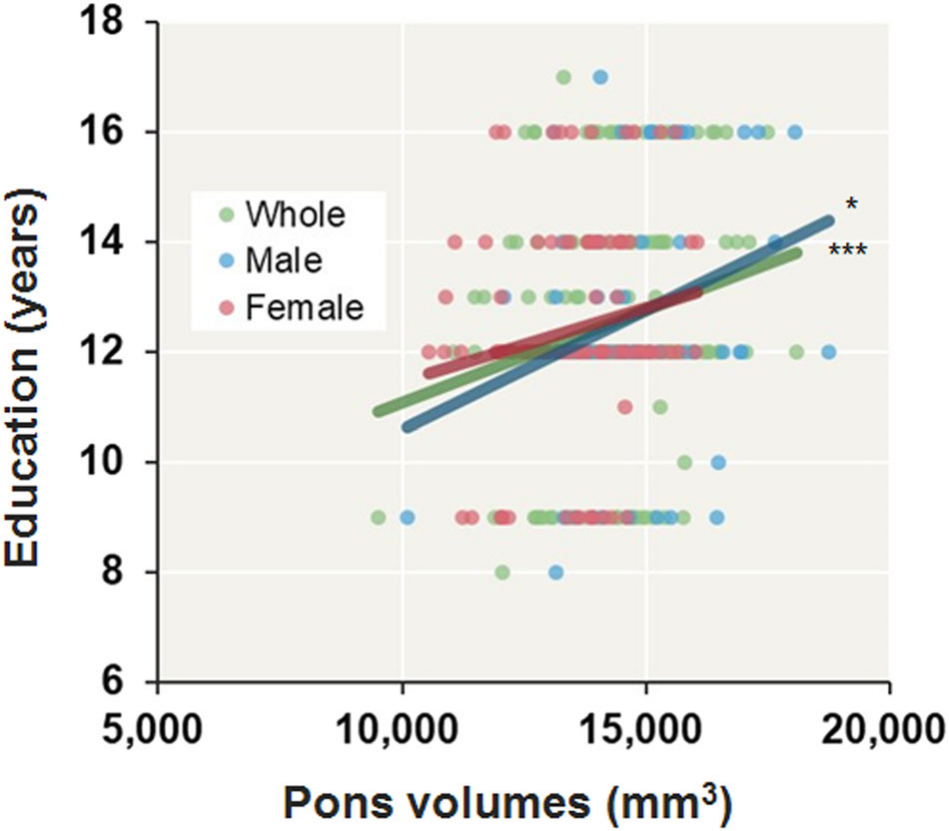
Correlations between years of education and age-, age-squared-, sex-, and ICV-corrected pons volumes in all patients, male patients, and female patients with schizophrenia. ^*^p < 0.05, ^***^p < 0.01.

Consistent with a previous study^3^, these brainstem volumes were not significantly correlated with current clinical symptoms in all patients (Supplementary Table 2) or in patients stratified by sex (*p* > 0.01). Of the other clinical variables (Supplementary Table 2), lower years of education were significantly correlated with smaller pons (*r* = 0.24, *p* = 3.06 × 10^−3^) and whole brainstem volumes (*r* = 0.24, *p* = 3.50 × 10^−3^) in patients (Fig. 2), particularly male patients (*p* < 0.05, pons, *r* = 0.31; whole brainstem, *r* = 0.29).

## Discussion

This is the first study to investigate sex differences in brainstem volumes between SZ patients and HCs. Among the brainstem structures, pons volumes were significantly reduced, particularly in male SZ patients. The decreased pons volumes were associated with lower levels of education in male patients.

Although we found a difference only in pons volumes between SZ patients and HCs among four brainstem structures, the effect sizes for the differences in brainstem volumes between the case and control groups were similar between the present and previous studies (Fig. 1). The effect sizes in this study were also moderate (*d* = −0.08 for midbrain to −0.25 for pons), except for SCP (*d* = −0.003).

The pons is involved in a variety of functions, such as the regulation of sleep/arousal, respiration, cranial nerves, and reflexes. It is currently not fully understood why pons volumes are reduced in SZ patients, particularly male SZ patients. However, some hypotheses might explain this finding. The reduction in pons volume may be due to a disruption in brain development during early life in SZ patients or a loss of neural cells or changes in the structure of the pons as a result of the disorder process. Considering the negative correlation of pons volume with education levels but not duration of illness particularly in male SZ patients and evidence that male patients have poorer premorbid adjustment than female patients^7^, the reduction in pons volume in male patients might be affected by changes before or around the onset of the disorder. Furthermore, as sex hormones such as estrogen can affect brain development^11^ and may modulate the expression of genes involved in SZ, hormonal differences may also play a role in sex differences in the brainstem volumes. The brainstem volume reductions could be related to the disorder’s genetic susceptibility^3^, hormonal factor and environmental factors, such as prenatal stress, infection, or substance use. The sex differences in brainstem volumes may cause sex differences in physiological function, behavior, cognition, symptom presentation and treatment response.

There are some limitations to the interpretations of our findings. Mean age and sex ratio significantly differed between SZ patients and HCs. Although we corrected for age, age-squared, sex and ICV as covariates, these confounding factors might influence our findings.

In summary, we observed decreased pons volumes particularly in male SZ patients, and the decreased pons volumes were related to low education levels. Further research is needed to understand the underlying causes and timing of the reduced pons volume in SZ.

## Supplementary information

Supplementary Information

## Data Availability

Our data are not publicly available because they contain information that could compromise the research participants’ privacy/consent.
