# Supplementary material for: Sex differences in brainstem structure volumes in patients with schizophrenia
Source: Schizophrenia (Heidelb). 2023 Mar 18;9(1):16. doi: 10.1038/s41537-023-00345-0 (PMC10024760; doi:10.1038/s41537-023-00345-0)

**Supplementary Information**

**Supplementary Table 1.** Demographic characteristics of patients with schizophrenia and healthy subjects stratified by sex.

|  | HCs | | SZ | |  |
| --- | --- | --- | --- | --- | --- |
|  | Male | Female | Male | Female | HCs *vs.* SZ |
| Variables | (*n*=133) | (*n*=72) | (*n*=61) | (*n*=95) | *p* values (*F* or *χ^2^*) |
| Sex (male/female) | 133/0 | 0/72 | 61/0 | 0/95 | **1.14×10^-6^ (23.7)^a^** |
| Age (years) | 35.5 ± 13.7 | 36.1 ± 13.4 | 41.0 ± 13.3 | 44.7 ± 13.2 | **2.29×10^-7^ (27.8)** |
| Education (years) | 16.7 ± 2.0 | 15.5 ± 2.8 | 12.6 ± 2.4 | 12.4 ± 2.0 | **2.64×10^-42^ (243.8)** |
| Estimated premorbid IQ | 102.7 ± 27.4 | 104.3 ± 23.6 | 99.0 ± 17.7 | 97.8 ± 10.9 | **0.035 (4.5)** |
| Present IQ | 108.7 ± 9.5 | 109.1 ± 11.4 | 85.0 ± 21.4 | 80.5 ± 15.5 | **4.10×10^-35^ (211.6)** |
| ICV (cm^3^) | 1570.4 ± 138.7 | 1446.6 ± 112.0 | 1518.9 ± 158.4 | 1425.2 ± 130.5 | **3.17×10^-5^ (17.8)** |
| Handedness (Rt/Lt) | 123/10 | 71/1 | 60/1 | 89/6 | 0.70 (0.1)^a^ |
| CPZ-eq (mg/day) | - | - | 473.4 ± 415.7 | 521.8 ± 570.0 | - |
| BPD-eq (mg/day) | - | - | 0.6 ± 1.5 | 1.0 ± 2.8 | - |
| Age at onset (years) | - | - | 25.5 ± 9.3 | 27.9 ± 12.1 | - |
| DOI (years) | - | - | 15.4 ± 12.1 | 16.8 ± 12.1 | - |
| PANSS positive symptoms | - | - | 16.3 ± 5.5 | 16.0 ± 6.4 | - |
| PANSS negative symptoms | - | - | 19.4 ± 7.1 | 17.7 ± 6.7 | - |

HCs, healthy controls; SZ, schizophrenia; IQ, intelligence quotient; ICV, intracranial volume; Rt, right; Lt, left; CPZ-eq, total antipsychotic dosage in chlorpromazine equivalents; BPD-eq, biperiden equivalents of total antiparkinsonian drugs; DOI, duration of illness; PANSS, Positive and Negative Syndrome Scale. Complete demographic information was not obtained for all subjects (estimated premorbid IQ in male HCs, *n*=130; female HCs, *n*=68; male SZ patients, *n*=58; female SZ patients, *n*=92; present IQ in male HCs, *n*=91; female HCs, *n*=43; male SZ patients, *n*=47; female SZ patients, *n*=70). Means ± SDs are shown. ^a^ *χ^2^* test. *P* values <0.05 are shown in boldface.

**Supplementary Table 2. Partial correlations between brainstem volumes and clinical variables with age, age-squared, sex, and ICV as covariates in all patients, male patients, and female patients with schizophrenia.**

|  | Midbrain | | | Pons | | | SCP | | | Medulla | | | Whole brainstem | | |
| --- | --- | --- | --- | --- | --- | --- | --- | --- | --- | --- | --- | --- | --- | --- | --- |
|  | *Whole* | *Male* | *Female* | *Whole* | *Male* | *Female* | *Whole* | *Male* | *Female* | *Whole* | *Male* | *Female* | *Whole* | *Male* | *Female* |
| Education (years) | **0.22^***^** | 0.23 | **0.22^*^** | **0.24^***^** | **0.31^*^** | 0.17 | 0.10 | 0.07 | 0.17 | 0.12 | 0.16 | 0.08 | **0.24^***^** | **0.29^*^** | 0.18 |
| Estimated premorbid IQ | 0.02 | 0.09 | -0.08 | 0.12 | 0.20 | <-0.01 | 0.01 | -0.05 | 0.04 | -0.05 | -0.06 | -0.09 | 0.08 | 0.14 | -0.03 |
| Present IQ | 0.09 | 0.27 | -0.08 | 0.09 | 0.23 | -0.03 | -0.01 | -0.09 | 0.07 | -0.01 | 0.09 | -0.07 | 0.08 | 0.23 | -0.05 |
| CPZ-eq (mg/day) | -0.08 | -0.18 | -0.03 | -0.07 | -0.06 | -0.10 | -0.13 | 0.09 | **-0.25^**^** | 0.02 | -0.07 | 0.05 | -0.06 | -0.09 | -0.07 |
| BPD-eq (mg/day) | 0.03 | 0.06 | 0.02 | 0.01 | 0.07 | <0.01 | -0.03 | **0.27^*^** | -0.16 | 0.10 | 0.16 | 0.11 | 0.03 | 0.10 | 0.02 |
| Age at onset (years) | <-0.01 | -0.11 | 0.07 | 0.03 | -0.15 | 0.14 | -0.03 | -0.21 | 0.05 | -0.08 | -0.13 | -0.05 | <0.01 | -0.16 | 0.11 |
| DOI (years) | <0.01 | 0.11 | -0.07 | -0.03 | 0.14 | -0.14 | 0.03 | 0.21 | -0.05 | 0.08 | 0.13 | 0.05 | <0.01 | 0.15 | -0.11 |
| PANSS positive symptoms | 0.06 | 0.05 | 0.06 | -0.03 | -0.18 | 0.04 | -0.01 | -0.10 | 0.04 | 0.09 | -0.05 | 0.16 | 0.01 | -0.12 | 0.07 |
| PANSS negative symptoms | -0.01 | 0.10 | -0.13 | 0.03 | 0.01 | -0.01 | 0.06 | 0.01 | 0.10 | **0.16^*^** | 0.14 | 0.11 | 0.05 | 0.06 | -0.01 |

ICV, intracranial volume; SCP, superior cerebellar peduncle; IQ, intelligence quotient; CPZ-eq, total antipsychotic dosage in chlorpromazine equivalents; BPD-eq, biperiden equivalents of total antiparkinsonian drugs; DOI, duration of illness; PANSS, Positive and Negative Syndrome Scale. The partial correlation coefficient *r* is shown. ^*^*p*<0.05, ^**^*p*<0.0125, ^***^*p*<0.01.

**Supplementary Figure 1.** Effect sizes (Cohen’s *d*) for the differences in brainstem volumes between patients with schizophrenia and healthy subjects among the present and previous studies. The effect sizes were calculated using the age-, age-squared-, sex-, and ICV-corrected brainstem volumes for the whole sample and age-, age-squared-, and ICV-corrected brainstem volumes for sex-specific samples.


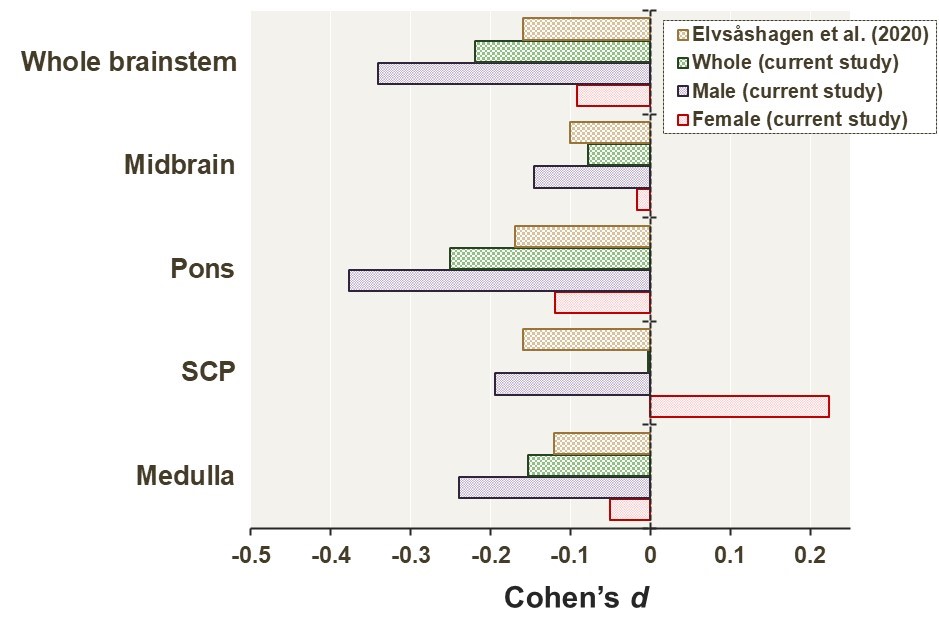

Supplement: Supplementary file 1 — Supplementary Information [file 41537_2023_345_MOESM1_ESM.doc]
